# Effectiveness of bimodal auditory and electrical stimulation in patients with tinnitus: A feasibility study

**DOI:** 10.3389/fnins.2022.971633

**Published:** 2022-08-24

**Authors:** Shikha Spencer, Marzena Mielczarek, Jurek Olszewski, Magdalena Sereda, Iris Joossen, Hanne Vermeersch, Annick Gilles, Sarah Michiels

**Affiliations:** ^1^Department of Otolaryngology, Laryngological Oncology, Audiology and Phoniatrics, Medical University of Lodz, Lodz, Poland; ^2^School of Medicine, Hearing Sciences, Mental Health and Clinical Neurosciences, University of Nottingham, Nottingham, United Kingdom; ^3^National Institute for Health and Care Research (NIHR) Nottingham Biomedical Research Centre, Nottingham, United Kingdom; ^4^Department of Otorhinolaryngology, Antwerp University Hospital, Edegem, Belgium; ^5^Department of Translational Neurosciences, Faculty of Medicine and Health Sciences, University of Antwerp, Antwerp, Belgium; ^6^Department of Education, Health and Social Work, University College Ghent, Ghent, Belgium; ^7^REVAL—Rehabilitation Research Center, Hasselt University, Diepenbeek, Belgium

**Keywords:** auditory, somatosensory, electrical, bimodal stimulation, treatment, tinnitus

## Abstract

**Background:**

Tinnitus is a common symptom, affecting about 10–15% of the adult population. When input from the somatosensory system can influence and/or elicit tinnitus, this type of subjective tinnitus is called somatosensory tinnitus. Recently, a new type of bimodal neurostimulation treatment has shown promising results for a specific subgroup within the somatosensory tinnitus population. It is, however, not clear if this bimodal stimulation is also effective in patients with other types of subjective tinnitus.

**Aim:**

The aim of this study was to evaluate the feasibility and efficacy of non-invasive bimodal auditory-somatosensory stimulation in reducing tinnitus severity among a general population of people with subjective tinnitus.

**Methods:**

Chronic subjective tinnitus patients were recruited from the ENT department of the Antwerp University Hospital. Somatosensory stimulation was delivered by Transcutaneous Electrical Nerve Stimulation (TENS), and it was combined with auditory stimulation *via* headphones. The therapy comprised six sessions of thirty minutes twice a week for a period of 3 consecutive weeks. Follow up measurements were scheduled 9–12 weeks after the last treatment session. The change of the Tinnitus Functional Index (TFI) score, a questionnaire evaluating tinnitus burden and effects on the quality of life, was the primary outcome measure.

**Results:**

Twenty-nine patients were enrolled in the study. A linear mixed-effects model was used to analyze the efficacy of bimodal treatment. The results of this analysis showed a statistically significant decrease (by 6, 9 points) in average TFI score at the follow up visit when compared to baseline. The ability to modulate tinnitus did not have an influence on the treatment results.

**Conclusion:**

Our study showed that bimodal stimulation is a feasible and safe method of tinnitus treatment. The method might be an effective treatment for some participants with tinnitus, especially those who have accompanying neck/temporomandibular problems, although, the evidence from this trial is quite weak. Additional research is needed toward establishing the optimal treatment protocol, as well as selecting the most appropriate inclusion criteria.

## Introduction

Tinnitus is defined as the perception of sound without the presence of any corresponding external stimuli. Patients describe the phenomenon as a ringing, buzzing, humming, or hissing perceived in the ear(s) or in the head. It can be pulsatile, non-pulsatile, continuous or intermittent (Han et al., 2009; Baguley et al., 2013). Globally, about 10–15% of the adult population experiences tinnitus (McCormack et al., 2016). While many of these patients habituate to the phantom sound, in around 1–2% of patients, tinnitus has a major impact on the quality of life. Those affected often suffer from sleep disorders, anxiety, and depression (Pattyn et al., 2016; Bhatt et al., 2017; Ziai et al., 2017; Geocze et al., 2018).

Tinnitus is a complex multifactorial condition which can be caused by pathological changes at any level of the auditory system (Zenner, 1998; Haider et al., 2018). Despite intensive research conducted in the past, the exact underlying mechanism of tinnitus generation is yet to be understood. One of the proposed mechanisms of tinnitus generation states that the phantom sound of tinnitus is generated as a result of compensatory events that occur after damage to cochlear hair cells (Noreña, 2015). The reduced motility of outer hair cells leads to deficit in the auditory signal conveyed to the central auditory system. To compensate for this reduced auditory signal, changes in inhibitory and excitatory activity occur in dorsal and ventral cochlear nuclei (Auerbach et al., 2014; Wu et al., 2016). Corresponding neurons in the dorsal and ventral cochlear nuclei reduce inhibitory activity by reducing release of inhibitory neurotransmitters including gamma-aminobutyric acid (GABA) and glycine (Auerbach et al., 2014; Lee and Godfrey, 2014; Caspary and Llano, 2017). These processes lead to the increase in the spontaneous firing rate which is further transmitted to inferior colliculi (IC). The IC project ascending fibers to medial geniculate body (MGB) of the thalamus. Therefore, neurons in the MGB also increase their spontaneous firing rate which is coherent in spatial and temporal aspect (this is also known as neuronal hypersynchrony) (Eggermont and Tass, 2015). The neuronal hyperactivity at the level of the MGB is further followed by neuroplastic changes in the auditory cortex (Caspary and Llano, 2017). In the auditory cortex, the neurons are arranged in an order to respond to specific frequencies of sound (known as tonotopic organization) (Wang et al., 2020). After the cascade of events following the decrease in auditory signal from the peripheral system, a reorganization of this tonotopic map is observed. The neurons corresponding to a certain frequency of sound in the tonotopic map start responding to the adjacent frequencies rather than responding to their primary frequencies, thereby reorganizing and extending the tonotopic map (tonotopic reorganization) (Mühlnickel et al., 1998). Thus, the hyperexcitability in terms of spontaneous neuronal firing in the resting state, abnormal neural synchrony and tonotopic reorganization in the auditory cortex are hypothesized to be major factors contributing to tinnitus generation and perception (Eggermont and Roberts, 2004; Adjamian et al., 2009; De Ridder et al., 2015; Haider et al., 2017, 2018).

Tinnitus is often associated with hearing loss (Tan et al., 2013), however, not all tinnitus patients suffer from hearing loss which suggests that non-auditory components also contribute to the mechanism of tinnitus generation. Animal research revealed neural connections between the auditory and somatosensory systems (Zhou and Shore, 2004; Shore, 2005; Shore et al., 2007). More specifically, the dorsal cochlear nucleus (DCN) has connections with the somatosensory brainstem nuclei receiving afferent information from the temporomandibular and upper cervical spine regions (Zhou and Shore, 2004; Shore, 2005). Therefore, the DCN is the site of multi-sensory integration (Basura et al., 2012; Shore and Martel, 2019). Through these connecting fibers, altered somatosensory input can cause abnormal activation in the DCN, resulting in the increased spontaneous firing rate and disturbed neuronal synchrony (Kaltenbach, 2006).

Somatosensory input from cervical spine or temporomandibular joint may influence the excitation or inhibition at the neuronal level, thereby influencing physiological correlates of tinnitus or even lead to tinnitus generation (Wright and Ryugo, 1996; Shore et al., 2007). The presence of these connections in humans has been demonstrated by Lanting et al. (2010), who found an increased activation of the auditory brainstem nuclei during active protrusion in patients with tinnitus who could modulate their tinnitus by jaw protrusion, compared to control subjects without tinnitus (Lanting et al., 2010). The normalization of this somatosensory input might contribute to the reduction in tinnitus loudness and severity and less excitation at the level of the cerebellum.

Transcutaneous electrical nerve stimulation (TENS) of the upper cervical spine or temporomandibular area is one possible way of altering somatosensory input in a non-invasive way. The technique has been widely used as a therapeutic modality for the treatment of acute and chronic pain syndromes (Bjordal et al., 2003; Johnson and Martinson, 2007; Zhu et al., 2017). Moller (2000) reported that electrical stimulation of the median nerve could modulate tinnitus (i.e., increase or decrease in loudness). Several studies investigating the efficacy of TENS of e.g., upper cervical spine (C2), mastoid, pre-auricular skin, auricle and tympanic membrane for reducing tinnitus severity have been conducted so far (Dobie et al., 1986; Steenerson and Cronin, 2003; Herraiz et al., 2007; Vanneste et al., 2010; Lee et al., 2014; Li et al., 2019). In general, findings from these studies revealed that tinnitus severity could potentially be decreased by TENS, however, only a small number of patients benefited from the treatment. Therefore, in the past decade a new treatment method has emerged, namely the bimodal stimulation. This therapy combines two types of stimuli (for example auditory stimuli and somatosensory stimuli) and aims to influence the auditory cortex at neuronal level, which can lead to suppression of tinnitus. The idea behind the use of the two different stimuli (engaging different neural pathways) is to increase the potential neuroplasticity by synchronizing the neural events (Hebb, 1949 in Morris, 1999). Especially, the combination of acoustic and somatosensory stimuli has received increasing interest after animal and human models explored the relationship between the auditory and somatosensory systems (Shore et al., 2016).

Several studies (De Ridder et al., 2014; Shim et al., 2015; Tyler et al., 2017; Marks et al., 2018; Conlon et al., 2020) previously investigated the efficacy of bimodal stimulation for tinnitus showing promising results. Marks et al. (2018), whose protocol we followed in the present study, showed that neural correlates of tinnitus can be modified by bimodal stimulation (auditory and somatosensory), but the effect depended on the precise order and timing between the two types of stimuli. In order to establish the most optimal protocol for tinnitus suppression, different stimulation conditions were explored in animal guinea pig model. The comparison of the stimulus order (auditory stimulus preceded somatosensory vs. somatosensory stimulus preceded auditory stimulus) showed that the first unit-pair weakened neural synchrony, whereas the second—strengthened it. Furthermore, the analysis of the three between stimulus intervals (5, 10, and 20 ms) revealed the best suppression of synchrony and spontaneous activity for 5 and 10 ms intervals, and slight changes for the 20 ms interval. However, when compared to unimodal auditory or somatosensory stimulation, 5 ms interval appeared to evoke significantly greater neuronal changes. In terms of the auditory stimulus, Marks et al. (2018) chose one possible concept, namely matching frequency of the sound to tinnitus spectrum. In result, a significant reduction of Tinnitus Index (quantifying behavioral signs of tinnitus in guinea pigs) was obtained at the treated frequency of 8 kHz (frequency at which tinnitus was the most prevalent) and not the other frequencies. Having in mind that human cochlear nucleus contains similar cellular elements as present in rodents’ DCN, the study protocol first determined in experimental animal studies was next applied in humans (Marks et al., 2018). A specific sample of patients with unilateral pure tone tinnitus that could be modulated during specific somatic maneuvers of the neck or jaw was recruited for this study. To obtain long-term depression, which was demonstrated to reverse decreased synchrony and spontaneous activity in fusiform cells and alleviate tinnitus, sound stimulus (tone-burst at tinnitus frequency) preceded electrical stimulus, with the stimulus interval of 5 ms. Somatosensory stimulus was delivered in C2 region in animals, in humans, however, the electrode was positioned on skin of the trigeminal ganglion region or C2 region, depending on which maneuvers induced the strongest tinnitus change. The clinically significant reduction of tinnitus [at least 13 points reduction in the Tinnitus Functional Index (TFI) score] was obtained in 10 out of 20 human participants receiving the active treatment (Marks et al., 2018).

The protocol applied in the current study was based on the study of Marks et al. (2018) who investigated that protocol in both animals and humans. For their human study, Marks et al. (2018) only included patients with a unilateral pure tone tinnitus that could be modulated by neck or jaw movements. However, this subgroup of patients with tinnitus is relatively small. It is probable that the used approach would also be effective in a larger group of tinnitus patients, as animal research has proven the presence of connections between the somatosensory and auditory system unrelated to the tinnitus subtype. Therefore, our study investigated the feasibility of applying the above protocol in a broader group of tinnitus patients.

Thus, the aim of this study was to evaluate the feasibility and efficacy of non- invasive bimodal auditory-somatosensory stimulation in reducing tinnitus severity in general subjective tinnitus population.

## Materials and methods

### Study design

This feasibility study was conducted at the Tinnitus Treatment and Research Centre Antwerp (TINTRA) at the ENT department of the Antwerp University Hospital, Belgium.

### Participants

Twenty-nine adult patients suffering from moderate to severe chronic subjective tinnitus (score less than 90 points on the THI) were recruited by a multidisciplinary team of otolaryngologists, audiologists and physiotherapists. During the consultation, patients were assessed and screened for eligibility considering the inclusion and exclusion criteria. Inclusion criteria were: (a) adult patients > 18 years, with chronic subjective tinnitus (defined as tinnitus duration of six months or more); (b) TFI score less than 90 points (patients for whom tinnitus is a serious problem should receive immediate help/treatment that is a current standard of care). Patients were excluded if they had any of the following: (a) objective or acute tinnitus (< 6 months duration); (b) tinnitus due to Meniere’s disease; (c) metal implants in the body; (d) pacemaker; (e) oncological conditions; (f) active middle ear pathologies; (g) severe hearing loss making the patient unable to hear the auditory stimulus used in the study; (h) skin lesions in region of neck and face (temporomandibular joint area).

The patients were informed about the treatment protocol and their written consent for the therapy was obtained before starting the treatment. The study protocol was approved by the Bioethics Committee of the Antwerp University Hospital (number B300201941421).

### Outcome measures

#### Primary outcome measure

TFI was used as the primary outcome measure. The TFI is a comprehensive scale, assessing tinnitus symptom severity, comprising 25 questions and it has shown good test-retest reliability (*r* = 0.75). It has eight subscales: intrusiveness, sense of control, cognitive, sleep, auditory, relaxation, quality of life, and emotional. In order to make sure that the reported results correspond to genuine change in tinnitus perception, we used a criterion of clinically relevant change. This means, following Meikle et al. (2012), a change in the total TFI score (increase or decrease) by at least 13 points, which correlates with clinically relevant improvement or clinically relevant deterioration in tinnitus. The other term used in the literature is clinically significant (Ranganathan et al., 2015) used to report the results which meant the noticeable change for the patient in the perception of tinnitus (keeping in mind that statistically significant change in the total TFI score might not be perceived by patient). The TFI was completed at a baseline, immediately after treatment and at follow up which is 9–12 weeks after the last treatment session.

#### Secondary outcome measures

Apart from the primary outcome measure, we collected data from five different secondary outcome measures in order to evaluate the tinnitus loudness, the presence of temporomandibular disorders (TMD), the presence and degree of neck dysfunction, the presence of anxiety or depression and personality characteristics.

Tinnitus loudness: The visual analogue scale (VAS) was used to assess subjective tinnitus loudness. Patients were asked to report the average loudness of tinnitus in the past week on a 100 mm line. The left end of the line was marked with zero indicating no tinnitus, while the right end was marked with 100, indicating maximum loudness of tinnitus (Adamchic et al., 2012).

Temporomandibular disorders (TMD): The TMD-pain screener is a short, reliable and valid instrument to indicate the presence of temporomandibular disorders, with a sensitivity and specificity of 0.95. It is a 6-item questionnaire related to pain and complaints from the orofacial region. The questionnaire has good internal consistency (α value of 0.93) and acceptable reliability (intra-class correlation coefficient 0.79). A score of 3 points or more indicates the presence of TMD (Gonzalez et al., 2011).

Neck pain: The Neck Bournemouth Questionnaire (NBQ) assesses the presence of neck pain and its impact on a patient’s wellbeing, together with professional and daily activities (Bolton and Humphreys, 2002). It consists of seven questions that are scored on an 11-point Likert scale. The test-retest reliability of the NBQ is moderate (intra-class correlation coefficient 0.65). The construct validity was acceptable with both the Neck Disability Index (*r*: 0.50) and the Copenhagen Neck Functional Index (*r*: 0.44). The effect size was found to be high (Cohen’s *d*: 1.67), which indicates that the NBQ is highly responsive to changes in cervical spine complaints (Bolton and Breen, 1999). A score of 14 points or more on the Neck Bournemouth Questionnaire is considered as a clinically significant neck complaint (De Hertogh et al., 2007).

Anxiety and depression: The Hospital Anxiety and Depression Scale (HADS) is used to screen for the presence of clinical anxiety and depression. It has fourteen questions divided into two subscales; seven addressing anxiety and seven addressing depression. Each question is scored from 0 to 3 points. A score of 8 or more on one of the subscales indicates clinically significant anxiety/depression (Zigmond and Snaith, 1983).

Hyperacusis: The Hyperacusis Questionnaire (HQ) was used to determine the presence of hyperacusis. The questionnaire comprises fourteen questions addressing patient’s hypersensitivity to sound. Patient’s response to these questions is rated on a 4-point Likert scale. For each question the patient chooses between four response options: “no” (0 points), “yes, a little” (1 point), “yes, quite a lot” (2 points), and “yes, a lot” (3 points). The total score ranges between 0 and 42. A total score greater than 28 indicates hyperacusis (Khalfa et al., 2002).

All secondary outcome measures were completed at baseline, immediately after the last treatment session (3 weeks) and at 9–12 weeks (follow-up).

### Intervention

All patients received six 30-min sessions of bimodal auditory and electrical stimulation over a period of 3 weeks. Two stimulation sessions were scheduled in a week, with an interval of minimum 1 day in between the sessions. During the treatment, the patient was lying in supine position on the treatment table with knees in light flexion to ensure a comfortable position.

Auditory stimulation: The tone burst used as the auditory stimulus was matched to the patient’s tinnitus frequency. In each case of uni- and bilateral tinnitus, the auditory stimulation was provided bilaterally *via* on-ear headphones. The loudness of the auditory stimulus was adjusted to a clearly audible but comfortable level as subjectively perceived by the patient.

The tinnitus frequency was obtained by the use of a pitch matching technique (forced choice method) which is the quantitative (matching tinnitus pitch and loudness) and qualitative description (pure tone vs. noise band) of the spectral characteristics of tinnitus. For this technique, a two-alternative forced choice procedure was used, using the contralateral ear as a reference ear. In cases where tinnitus was perceived bilaterally, a reference ear was chosen randomly. By this technique, an attempt was made to identify the center pitch of tinnitus. When multiple tinnitus sounds were perceived, it was suggested to concentrate on the most troublesome tinnitus sound. Each time a pair of pure tones (or noises in case of noise-like tinnitus), differing by one or more octaves, were presented to the subject who had to indicate which of the tones resembles tinnitus the most. This procedure was repeated and finer adjustments were made to obtain a match of tinnitus pitch as exact as possible (Gilles et al., 2016).

Electrical stimulation: Somatosensory stimulation was provided using a portable TENS-device (EMPI TENS, Chattanooga) which was approved according to standard EN 60601-1 “Medical electrical equipment.” A high frequency burst-TENS was used at 150 Hz with an intensity adjusted to a clearly tangible but non-painful sensation. Self-adhesive electrodes were used to apply the electrical stimulation. The location of these electrodes was adjusted according to the patients’ ability to modulate tinnitus with maneuvers of jaw or neck. Electrodes were placed on the skin either (i) bilaterally next to the spinal process of C2 (C2-setup) or (ii) one electrode was positioned unilaterally on the temporomandibular joint (over the trigeminal ganglion) while the second electrode was positioned ipsilaterally next to the spinal process of C2 (TMJ-setup). The C2-setup was similar in case of unilateral and bilateral tinnitus. In case of tinnitus modulation with jaw maneuvers, the electrodes were placed in the TMJ-setup and in case of tinnitus modulation with neck movements, the C2-setup was used. When patients were not able to modulate their tinnitus, the TMJ-setup was used. In case of unilateral tinnitus, the TMJ- setup was placed at the tinnitus side. In case of bilateral tinnitus, the TMJ—setup was placed at the right side.

The timing of auditory and electrical stimulation was chosen in accordance with the study of Marks et al. (2018). The auditory stimulus consisted of a series of pure tone stimuli with 10 ms duration and 1 ms linear rise and fall time. The auditory stimulus was then combined with the electrical stimulus, where each auditory stimulus was followed by an electrical stimulus with a delay of 5 ms.

### Statistical analysis

Data was analyzed using SPSS^®^ vs. 24 and R. Intention to treat analysis was applied for the primary outcome measure (TFI). Only completed data were analyzed for secondary outcome measures. First, the normality of the data was investigated using a Kolmogorov-Smirnov test. Baseline comparability (*p* > 0.05) was analyzed using descriptive statistics, Mann-Whitney *U*-tests for non-normally distributed data and independent samples *t*-tests for normally distributed data. Chi square test was used to determine differences between dichotomous variables. To evaluate the change in total TFI score and the existence of a significant relationship at baseline between the total TFI score and the total NBQ score, HADS anxiety, and HADS depression, a linear mixed model was used. This analysis also evaluated the influence of time (i.e., baseline visit, immediately after treatment and follow up visit) on the total TFI score. Furthermore, this analysis reduced the variability introduced into the model by individualizing our patients. The effect of bimodal treatment on secondary outcome measures was evaluated using paired sample *t*-test comparing the outcome measures at baseline, immediately after treatment and at follow up.

## Results

### Patient flow and baseline characteristic

**FIGURE 1:**
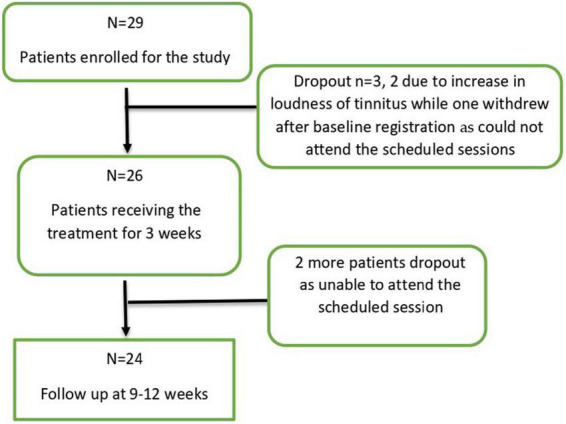
Consort flow diagram for patients’ enrollment and data analysis.

In total 29 patients with a mean age of 54.76 years (*SD* = 11.28; range: 26–70 years) were included in this study. Three out of 29 patients discontinued the study (two due to an increase in the intensity of tinnitus and one could not attend the scheduled sessions) thus a total of 26 patients completed the treatment sessions (*n* = 26). At follow up, two more patients dropped out being unable to attend the scheduled session, thus 24 participants finished the entire study protocol (Figure 1). An overview of the baseline patient characteristics can be found in Table 1.

**TABLE 1:** Effect of treatment on primary and secondary outcome measures.

Outcome measure	Baseline	Immediately after treatment	Follow up	Baseline—immediately after treatment	Baseline—follow up
**TFI**					
*N* Range Mean (*SD*)	29 9–70 46.7 (17.9)	26 10–81 47.2 (19.3)	24 5–72 41.1 (18.3)	β = −1.44, 95%CI [−5.49, 2.55], *p* = 0.497; Linear mixed-effects model*	β = −6.90, 95%CI [−1.94, −1.93], *p* = 0.012*; Linear mixed-effects model*
**HADS depression**					
*N* Range Mean (*SD*)	29 0–14 4.8 (4.0)	25 1–2 5.2 (3.6)	24 0–14 5.2 (3.6)	*t*(24) = −0.76, *p* = 0.45 (paired *t*-test)	*t*(23) = −1.56, *p* = 0.13 (paired *t*-test)
**HADS anxiety**					
*N* Range Mean (*SD*)	29 2–3 6.3 (2.8)	25 3–12 6.4 (2.3)	24 3–15 6.3 (3.2)	*t*(24) = 0.09, *p* = 0.93 (paired *t*-test)	*t*(23) = −0.20, *p* = 0.84 (paired *t*-test)
**HQ**					
*N* Range Mean (*SD*)	29 6–41 20.0 (8.4)	25 6–36 18.0 (8.1)	24 3–64 21.5 (14.7)	*t*(24) = 2.28, *p* = 0.032* (paired *t*-test)	*t*(23) = −0.78, *p* = 0.44 (paired *t*-test)
**VAS (left ear)**					
*N* Range Mean (*SD*)	28 0–100 54.8 (24.2)	22 5–100 54.3 (24.4)	19 10–90 57.7 (23.2)	*t*(21) = 0.25, *p* = 0.81 (paired *t*-test)	*t*(18) = 0.19, *p* = 0.85 (paired *t*-test)
**VAS (right ear)**					
*N* Range Mean (*SD*)	26 7–100 49.8 (21.9)	22 0–100 49.8 (27.5)	19 1–90 49.4 (27.4)	*t*(21) = 0.11, *p* = 0.91 (paired *t*-test)	*t*(18) = 0.29, *p* = 0.77 (paired *t*-test)
**NBQ**					
*N* Range Mean (*SD*)	29 0–51 11.5 (13.1)	25 0–49 14.0 (13.1)	14 0–26 9.4 (7.3)	*t*(24) = −0.78, *p* = 0.44 (paired *t*-test)	*t*(13) = −0.28, *p* = 0.78 (paired *t*-test)
**TMD pain screener**					
*N* Range % of TMD	16 0–4 3.4	16 0–4 3.7	16 0–5 4	x^2^(2) = 4, *p* = 0.135 (Friedman test for the overall effect of the treatment)	

*Estimates are adjusted for NBQ, HADS depression, and HADS anxiety. SD, standard deviation; VAS, visual analog scale; HADS, Hospital Anxiety and Depression scale; TMD, temporomandibular disorders; NBQ, Neck Bournemouth Questionnaire; HQ, hyperacusis questionnaire.

Six out of 29 patients included in the study were able to modulate their tinnitus. The visual inspection of the characteristics revealed no obvious trends among them and a small size of this subgroup was insufficient to perform any follow up analyses taking this factor into consideration. For comparison with the previous study by Marks et al. (2018) we have used asterisk sign to mark these patients in Supplementary Table 1.

### Adverse events

The method of non-invasive bimodal stimulation applied in our study was tolerated well. There were no cases of severe adverse events. Considering entire time of the study (from enrollment until follow up visit) the overall drop out ratio appeared quite high (5 out of 29 patients included initially in the study), but among them only two patients discontinued the study due to increase in tinnitus.

Directly after the therapy, there were 4 patients for whom their TFI score increased (clinically significant increase of at least 13 points). However, at the follow up there was only one person (out of 24) with increase in the TFI score of 13 points or more. The increase in the TFI scores could have resulted from the therapy itself, however, some other factors like increased stress, too much focus on tinnitus, which were not directly linked to the therapy, could have led to such increase.

### Reduction in tinnitus severity

The analysis of the linear mixed model evaluating the efficacy of bimodal treatment showed a statistically significant decrease in total TFI score (by 6.9 points) at the follow up (9–12 weeks, mean = 41.14, *SD* = 18.30) *p* < 0.05, but not immediately after treatment (mean = 47.15, *SD* = 19.30) when compared to baseline data (mean = 46.71, *SD* = 17.89) (Table 1).

This effect was significant, even when controlling for NBQ total, HADS anxiety, and HADS depression at baseline. The value from the TFI questionnaire is lower by 6.9 points at the follow up visit when compared to the visit prior to receiving the treatment. Based on the results of the model, it can also be concluded that patients during the follow up visit had a significantly better treatment outcome than the patients prior to receiving the treatment.

The mixed linear effect model was used to evaluate the potential existence of correlation between baseline total NBQ score, HADS anxiety, and HADS depression scores and total TFI score with respect to time of the visit (i.e., immediately after treatment and at follow up). The results of this analysis showed that these factors did not have any significant correlation with total TFI score (baseline total NBQ and TFI score, *p* = 0.074, baseline HADS anxiety and TFI score *p* = 0.140, baseline HADS depression and TFI score *p* = 0.183) (Table 2).

**TABLE 2:** Mixed effect linear regression model explaining the change in the TFI questionnaire results.

	Estimate	2.5%	97.5%	*P*-value
Intercept	32.241	22.765	41.961	<0.001
Total TFI score post treatment	−1.442	−5.494	−2.545	0.497
Total TFI score at follow up	−6.903	−1.935	−1.931	0.012
Baseline total NBQ	0.267	−0.012	0.546	0.074
Baseline HADS anxiety	1.187	−0.319	2.692	0.140
Baseline HADS depression	0.833	−0.438	2.088	0.183

*Visit prior to treatment is used as reference level. HADS, Hospital anxiety and depression scores. NBQ, Neck Bournemouth Questionnaire; TFI, Tinnitus Functional Index.*

Applying the criterion of clinically relevant change in tinnitus (at least 13-point increase or decrease in total TFI score), immediately after treatment, 2 out of 26 patients reported improvement in tinnitus and 4 out of 26 patients reported increase in tinnitus severity (the tinnitus deterioration was not maintained at follow up). At follow up 6 out of 24 patients reported improvement (they were not the same patients who improved immediately after treatment), while 1 patient reported increase in tinnitus severity. Out of the six participants with clinically significant improvement at follow up, 5 experienced tonal tinnitus and 1 had noise like tinnitus.

### Effect on secondary outcome measures

There were no significant differences immediately after treatment or follow-up except for a statistically significant reduction in HQ score immediately after treatment [*t*(24) = 2.28, *p* = 0.032, paired *t*-test] (Table 1).

In general, there were six individuals in whom NBQ was high at baseline (above 14 points). Among these six participants, five achieved clinically significant improvement in TFI (2 at immediately after treatment and 3 at follow up), while one did not achieve clinically significant improvement.

### Characteristics of clinically relevant improvers

The visual inspection of the characteristics showed that the two out of twenty-six (2/26) patients who reported clinically relevant improvement on TFI immediately after treatment, suffered from a high degree (above 14 points) of neck complaints measured with NBQ (44 and 51 points at baseline respectively; a score of 14 points or more means a clinically significant neck complaint (De Hertogh et al., 2007). The first patient (BT25; 26 years old with noise tinnitus) was able to modulate the tinnitus during the somatic neck maneuvers we applied. In this case we observed a substantial NBQ reduction (from 44 to 25) after treatment. Such a pronounced reduction was not observed in the second patient (BT16, 51 years old, tonal tinnitus; NBQ decrease from 51 to 48 points). In BT25 improvement in TFI immediately after the treatment was not maintained at follow up, BT16, however, did not appear at follow up assessment thus we cannot be sure if the improvement was maintained (Supplementary Table 2).

The visual inspection of the patient characteristics at follow- up showed that among 6 clinically relevant improvers, there were 4 patients in whom a complete (BT01 from 23 to 0, BT08 from 20 to 0, BT23 from 4 to 0) or substantial reduction (BT17 from 42 to 26) of NBQ scores was observed. One participant (BT01) could modulate the tinnitus with jaw movements (see Supplementary Table 3 for detailed characteristics). Due to the small number of improvers in the current study, subgroup analyses were not conducted.

## Discussion

The aim of our study was to evaluate the feasibility and efficacy of non-invasive bimodal auditory-somatosensory stimulation in reducing tinnitus severity among a general population of patients with subjective tinnitus. Bimodal stimulation resulted in a statistically significant improvement in tinnitus severity (average TFI score reduction of 6, 9 points) at follow up but not immediately after treatment. However, when we consider the criterion of clinically relevant improvement in tinnitus (defined as reduction in total TFI score by at least 13 points) only 2 out of 26 patients reported improvement immediately after the treatment and 6 out of 24 patients at follow up.

We reported both the statistically significant improvement and clinically relevant improvement (Meikle et al., 2012) to describe the effects of bimodal stimulation on tinnitus symptom severity measured with TFI. While statistically significant improvement suggested that the bimodal treatment might have a potential to improve tinnitus in a general population of patients with subjective tinnitus, only small proportion of those patients could actually perceive the improvement in their tinnitus (TFI score improvement by 13 or more points). Therefore, while the results seem promising, more work should be done to adjust the treatment protocol or treatment intensity (e.g., more sessions) to achieve clinically significant improvement in larger proportion of participants.

The method applied in our study—non-invasive bimodal auditory-somatosensory stimulation, similarly to other studies (Shim et al., 2015; Marks et al., 2018; Conlon et al., 2020) was tolerated well, with no occurrence of severe adverse events. We must keep in mind, however, the two patients who dropped out before the treatment completion due to reported increase in their tinnitus, which could have resulted from the actual increase in tinnitus loudness or the nocebo effect.

The delayed improvement at follow- up, rather than immediately after treatment, can potentially be explained by the concept of the slow process of neuroplasticity (Engineer et al., 2011; Markovitz et al., 2015; Sathappan et al., 2019). Neuroplastic effects develop at the central level following repetitive bimodal treatment sessions and thanks to combining two methods of stimulation, a cumulative effect could be obtained at follow up.

### Potential predictors of outcome

#### Patient characteristics

In order to select potential predictors of outcome, we used different secondary outcome measures. We analyzed the treatment results for possible correlations between tinnitus improvement (in TFI) and neck symptoms (in NBQ), the ability to modulate tinnitus with neck or jaw movements, anxiety and depression (in HADS). The statistical analysis revealed no significant correlations, thus no apparent predictors of outcome.

In the literature, the studies vary in the inclusion/exclusion criteria for auditory-somatosensory stimulation for tinnitus treatment, thus studied groups differ between the studies (Tyler et al., 2017; Marks et al., 2018; Conlon et al., 2020). The researchers, however, analyzed these populations in attempt to select some predictors of outcomes, which would serve to establish the optimal eligibility criteria for bimodal stimulation in the future.

The results of the specific treatment conditions (methods and parameters) may depend on specific patient/tinnitus aspects. For example, Tyler et al. (2017) using vagus nerve stimulation (VNS) paired with tones (excluding tinnitus spectrum) in sensorineural tinnitus patients, recognized greater benefits in participants who didn’t have hissing and/or blast-induced tinnitus (Tyler et al., 2017). On the other hand, Conlon et al. (2020) using sounds paired with tongue stimulation in chronic subjective tinnitus patients (but excluded somatic tinnitus caused by a head or neck injury) reported a trend toward greater improvement in TFI and THI for those who had worse tinnitus symptoms at baseline. The authors investigated the influence of different stimulation parameters on tinnitus severity, not individual difference between the studied subjects (Conlon et al., 2020).

The study by Marks et al. (2018), which protocol we used in the current study, obtained a clinically significant improvement in 50% of patients with unilateral pure tone tinnitus that could be modulated by somatic maneuvers. The fact that this specific population was included in the study was considered by the authors as a study limitation (it remained unknown whether the results would translate to other subgroups of tinnitus patients) (Marks et al., 2018). This led to designing the current study in general tinnitus population, using similar stimulation parameters. It is worth noticing, that in our study in 5 out of 6 clinically improved patients at follow up tinnitus was tonal, and in 4 out of 6 clinically improved patients, there was a pronounced reduction in the NBQ score at follow up assessment. Thus, it could suggest that tinnitus patients with tonal tinnitus and the neck symptoms might be more likely to benefit from auditory-somatosensory stimulation. However, due to the small number of improvers we were not able to explore this potential predictor of treatment outcome further. From studies done in the past it is known that the specific subgroup of somatic tinnitus patients, takes advantage in terms of reduction in tinnitus, from treating neck symptoms (Bechter et al., 2016; Michiels et al., 2016, 2017, 2019a,b; Sajadi et al., 2019; van der Wal et al., 2020). Apart from a few works [(Conlon et al., 2020, who considered TMJ disorder an exclusion criterion, or Marks et al., 2018) who treated somatic tinnitus patients] the subgroup of somatic tinnitus appears to be not enough addressed in bimodal stimulation.

In our study the ability to modulate tinnitus did not seem to have a predictive value for the treatment results. However, the proportion of clinically relevant improvers among those patients who were able to modulate their tinnitus in the current study (2 out of 6) and in the study by Marks et al. (2018) (10 out of 20) seems similar.

A study by Sathappan et al. (2019) suggested another patients’ characteristic to take into consideration: the hearing status. They demonstrated increased and/or redistributed projections from the trigeminal system to the cochlear nucleus after hearing loss (Sathappan et al., 2019). Thus, potentially the increased reactivity of the connections between the auditory and somatosensory systems after cochlear damage (hearing loss) could mean that patients with sensorineural hearing loss will be more responsive to somatosensory input when treated with bimodal stimulation. This, however, needs to be further investigated in human subjects. In the current study both normal and hearing-impaired patients were included, which might have led to less pronounced improvement in tinnitus severity.

#### Parameter characteristics

Animal and human research showed that combining the two inputs, e.g., auditory and somatosensory stimuli can lead to long term neuroplasticity in the auditory system and improve tinnitus (Marks et al., 2018; Conlon et al., 2020). Using similar to Marks et al. (2018) protocol (5 ms delay of somatosensory stimulus which followed auditory stimulus matched to tinnitus spectrum) we obtained statistically significant TFI score reduction at follow up, but the number of clinically relevant improvers was lower (6 in 24 patients at follow up in our study and 10 out of 20 in Marks et al., 2018 study). This can be the result of differences in studied populations (see above) or less intensive treatment protocol (in general 3 h of stimulation in our study vs. 14 h in Marks et al., 2018 study).

Conlon et al. (2020) used three different stimulation conditions (spectra of pure tones ranging from 100 to 8,000 Hz and interstimulus delays ranging from 0 to 950 ms) and concluded that at long-term assessment the higher-frequency tones with synchronized or shorter delayed tongue stimulation were more effective comparing to low-frequency and long delayed tongue stimulation (Conlon et al., 2020). What’s more, the repeated stimulation over longer period of time (1 h per day for 12 weeks, at minimum 36 h) may be the key factor leading to long-lasting changes in the brain, which are responsible for tinnitus improvement. Authors suggested that in order to avoid habituation effects, the stimulation settings should be varied over the course of treatment, e.g., after 6 weeks (Conlon et al., 2020). When interstimulus delay is concerned, Marks et al. (2018) animal study results are in line with Conlon et al. (2020) study, namely shorter delays (5 and 10 ms) appeared to more effectively induce long term depression thus reduce tinnitus (Marks et al., 2018). Thus, in our study we chose short interstimulus delay (5 ms) as a premise to reduce tinnitus more effectively.

In order to develop habituation to tinnitus and to induce neuroplastic changes at the level of central auditory system, Jastreboff’s neurophysiological model suggests to use the auditory stimuli which spectrum is similar to tinnitus spectrum (Jastreboff et al., 1996). Such an approach was applied in the current study and resulted in statistically significant reduction in the averaged TFI score but clinically relevant improvement was achieved only in 6 out of 24 patients. Another approach is sound stimulation with the use of frequency spectrum excluding tinnitus frequency. The postulated result of application of the notch filters sound is an inhibition of frequencies within the notch of tinnitus spectrum *via* lateral inhibition (Pantev et al., 2012). Tyler et al. (2017) approach (auditory stimulation with the use of frequencies surrounding the tinnitus spectrum, thus excluding tinnitus frequency) resulted in clinically meaningful improvement in THI in 50% of participants (8 out of 16), but Shim et al. (2015) using notched music paired with transcutaneous vagus nerve stimulation (tVNS) of the external ear, obtained no significant changes in tinnitus severity measured with THI. In conclusion, there seems to be no apparent advantage of using one approach over the other and both need further exploration.

### Future implications

Our study showed the feasibility and safety of bimodal auditory-somatosensory stimulation in general group of subjective tinnitus patients. However, since the improvement was not as pronounced as expected, selecting the proper inclusion criteria (e.g., somatic tinnitus, with neck or TMJ symptoms) seems crucial for future studies. Based on the literature and our research we can hypothesize that patients with somatic tinnitus might show a better improvement in tinnitus severity when compared to patients who do not present somatic influence on tinnitus. Other factors to be taken in consideration are tinnitus type (noise vs. tonal), the hearing status of the participants, intensity of the treatment protocol (number and duration of stimulation sessions) and/or the type of auditory stimulus in relation to tinnitus sound. To avoid confounding placebo effect, the study needs control group, ideally with placebo intervention (or active control).

### Study limitations

One limitation of our study is a small sample size, the other—lack of control group to assess the potential placebo effect. Having in mind our results (6 clinically relevant improvers out of 24 subjects at follow up), especially in the light of the results in the control group in Tyler et al. (2017) study (the improvement in 4 out of 14 patients, VNS unpaired from tones) or sham stimulation group in Marks et al. (2018) study (the improvement in 4 out of 10 participants, unimodal—auditory stimulation), we interpret the results with caution. Furthermore, the small number of clinically relevant improvers in our study, did not allow further analysis and selection of predictors of outcomes.

## Conclusion

Our study showed that bimodal stimulation is a feasible and safe method of tinnitus treatment. The method might be an effective treatment for some participants with tinnitus, especially those who have accompanying neck/TMJ problems, although, the evidence from this trial is quite weak. Additional research is needed toward establishing the optimal treatment protocol, as well as selecting the most appropriate inclusion criteria.

## Data availability statement

The original contributions presented in the study are included in the article/Supplementary material, further inquiries can be directed to the corresponding author/s.

## Ethics statement

The studies involving human participants were reviewed and approved by the Bioethics Committee of the Antwerp University Hospital (number B300201941421). The patients/participants provided their written informed consent to participate in this study.

## Author contributions

SM and AG designed the study protocol. SS performed data collection. SS, MM, and JO drafted the manuscript. SM, MS, and AG provided critical feedback on the manuscript. IJ and HV coordinated the clinical trial. All authors have read and agreed to the published version of the manuscript.
