# Supplementary material for: Effectiveness of bimodal auditory and electrical stimulation in patients with tinnitus: A feasibility study
Source: Front Neurosci. 2022 Aug 24;16:971633. doi: 10.3389/fnins.2022.971633 (PMC9449838; doi:10.3389/fnins.2022.971633)
Supplement: Supplementary file 1 [file Table_1.docx]

Supplementary Material

Supplementary Material

Table S1: Changes in TFI score from baseline to immediately after treatment and baseline to follow up for individual participants. BL=baseline, FU=follow up, change in TFI BL vs immediately after treatment = difference in total TFI score, baseline score compared with immediately after treatment, change in TFI BL vs FU= difference in total TFI score, baseline score compared to 9-12 week follow up. Single asterisk sign (*) indicates that tinnitus was modulated by neck movements and double asterisk (**) indicates that tinnitus was modulated by jaw movements.

| **Patient ID** | **Type of tinnitus** | **TFI score at BL** | **TFI score immediately after treatment** | **TFI score at FU** | **Change in TFI**  **BL vs immediately after treatment** | **Change in TFI**  **BL vs FU** |
| --- | --- | --- | --- | --- | --- | --- |
| BT01** | Noise | 38.8 | 36.4 | 21.6 | -2.4 | -17.2 |
| BT02** | Tone | 24.4 | 16 | 13.2 | -8 | -11.2 |
| BT03 | Tone | 42.4 | Didn’t complete | Didn’t complete | N/A | N/A |
| BT04 | Noise | 25.6 | 18.8 | 15.6 | -6.8 | -10 |
| BT05* | Tone | 49.6 | 45.6 | 57.6 | -4 | +8 |
| BT06** | Tone | 61.2 | 76.8 | 71.6 | +15.6 | +10.4 |
| BT07 | Noise | 57.6 | 56.8 | 45.2 | -0.8 | -12.4 |
| BT08 | Tone | 46.8 | 34 | 26 | -12.8 | -20.8 |
| BT09 | Tone | 32.8 | 37.2 | 53.2 | 4.4 | +20.4 |
| BT10 | Noise | 34 | 44 | 29.6 | +10 | -4.4 |
| BT11 | Tone | 69.6 | 67.6 | 63.6 | -2 | -6 |
| BT12 | Tone | 42 | 60 | 38.8 | +18 | -3.2 |
| BT13 | Tone | 8.8 | 10.4 | 18.4 | +1.6 | +9.6 |
| BT14 | Noise | 63.6 | 67.6 | 66 | +4 | +2.4 |
| BT15 | Tone | 65.2 | 58.4 | 50 | -7 | -15.2 |
| BT16 | Tone | 68.4 | 44.8 | Didn’t complete | -20.6 | N/A |
| BT17 | Tone | 65.2 | 55.2 | 48.4 | -10 | -16.8 |
| BT18 | Tone | 19.2 | 22 | Didn’t complete | +2.2 | N/A |
| BT19 | Tone | 43.3 | 43.6 | 36 | +0.3 | -7.3 |
| BT20* | Tone | 53.2 | 74 | 63.6 | +20.8 | -10.4 |
| BT21 | Tone | 43.6 | 46 | 55 | +2.4 | +9 |
| BT22 | Noise | 21.6 | Didn’t complete | 29.2 | N/A | +7.6 |
| BT23 | Tone | 68 | 66.4 | 51.2 | -1.6 | -16.8 |
| BT24 | Tone | 17.2 | 21.6 | 48.4 | +4.4 | -12 |
| BT25* | Noise | 59.6 | 44.4 | 52 | -15.2 | -7.6 |
| BT26 | Tone | 63.3 | 81.2 | Didn’t complete | +17.9 | N/A |
| BT27 | Tone | 42 | 43.2 | 37.2 | -1.2 | -4.8 |
| BT28 | Tone | 62 | 54 | 39.2 | -8 | -22.8 |
| BT29 | Tone | 65.6 | Drop out | Drop pout | Drop out | Drop out |

Table S2: Clinically relevant improvers. Effect on secondary outcome measures from baseline to immediately after treatment. BL- baseline, NBQ - Neck Bournemouth Questionnaire, HADS - Hospital Anxiety and Depression Scale

| **Patient ID / age** | **Total TFI score**  **at BL** | **Total TFI score immediately after treatment** | **HADS Anxiety score**  **at BL** | **HADS Depression score at BL** | **HADS Anxiety score immediately after treatment** | **HADS Depression score immediately after treatment** | **NBQ score**  **at BL** | **NBQ score immediately after treatment** | **Hyperacusis score at BL** | **Hyperacusis score immediately after treatment** |
| --- | --- | --- | --- | --- | --- | --- | --- | --- | --- | --- |
| BT16/ 51 | 68.4 | 44.8 | 10 | 14 | 6 | 9 | 51 | 48 | 35 | 23 |
| BT25*/ 26 | 59.6 | 44.4 | 10 | 4 | 6 | 2 | 44 | 25 | 21 | 16 |

*An improver able to modulate tinnitus with neck movements.

Table S3: Clinically relevant improvers. Effect on outcome measures at 9-12 week follow up. BL- score at baseline, FU- score at 9 week follow up, HYP- Hyperacusis score, NBQ - Neck Bournemouth Questionnaire, HADS - Hospital Anxiety and Depression Scale. Improver able to modulate tinnitus with *neck or ** jaw movements. Baseline is used as reference level, estimates =correlation estimates.

| **Patient**  **ID / age** | **Total TFI score BL** | **Total TFI score immediately after treatment** | **Total TFI score at FU** | **HADS Anxiety score at BL** | **HADS Anxiety score FU** | **HADS Depression score at BL** | **HADS Depression score at FU** | **Total NBQ score at BL** | **Total NBQ score at FU** | **HYP BL** | **HYP FU** |
| --- | --- | --- | --- | --- | --- | --- | --- | --- | --- | --- | --- |
| BT01**/ 63 | 39 | 36 | 22 | 4 | 5 | 1 | 1 | 23 | 0 | 13 | 9 |
| BT08/ 28 | 47 | 34 | 26 | 5 | 5 | 3 | 3 | 20 | 0 | 24 | 34 |
| BT15/ 53 | 65 | 58 | 50 | 2 | 5 | 4 | 8 | 0 | 8 | 21 | 20 |
| BT17/ 56 | 65 | 55 | 48 | 4 | 5 | 4 | 7 | 42 | 26 | 18 | 22 |
| BT23 / 63 | 68 | 66 | 51 | 8 | 3 | 5 | 6 | 4 | 0 | 10 | 3 |
| BT28 /58 | 62 | 54 | 39 | 6 | 8 | 4 | 8 | 5 | 8 | 28 | 28 |
